# Anti-Th/To Antibodies in Scleroderma: Good Prognosis or Serious Concern?

**DOI:** 10.3390/jcm13113022

**Published:** 2024-05-21

**Authors:** Maria Możdżan, Andrzej Węgiel, Laura Biskup, Olga Brzezińska, Joanna Makowska

**Affiliations:** Department of Rheumatology, Medical University of Lodz, 90-549 Lodz, Poland; andrzej.wegiel@stud.umed.lodz.pl (A.W.); laura.biskup@stud.umed.lodz.pl (L.B.); olga.brzezinska@umed.lodz.pl (O.B.)

**Keywords:** scleroderma, anti-Th/To, autoantibodies, RNAse MRP and P, interstitial lung disease, pulmonary arterial hypertension, systemic sclerosis

## Abstract

Systemic sclerosis (SSc) represents a rare and intricate autoimmune connective tissue disease, the pathophysiology of which has not been fully understood. Its key features include progressive fibrosis of the skin and internal organs, vasculopathy and aberrant immune activation. While various anti-nuclear antibodies can serve as biomarkers for the classification and prognosis of SSc, their direct role in organ dysfunction remains unclear. Anti-Th/To antibodies are present in approximately 5% of SSc patients, and are particularly prevalent among those with the limited subtype of the disease. Although the presence of these autoantibodies is associated with a mild course of the disease, there is a strong connection between them and severe clinical manifestations of SSc, including interstitial lung disease, pulmonary arterial hypertension and gastrointestinal involvement. Also, the additional clinical correlations, particularly with malignancies, need further research. Moreover, the disease’s course seems to be influenced by antibodies, specific serum cytokines and TLR signaling pathways. Understanding the relationships between presence of anti-Th/To, its molecular aspects and response to treatment options is crucial for the development of novel, personalized therapeutic techniques and should undergo profound analysis in future studies.

## 1. Background

Systemic sclerosis (SSc), also known as scleroderma, is a chronic autoimmune disorder with diverse symptoms [1]. Its pathogenesis is based on three main factors: an excessively activated immune system, endothelial cell activity and fibroblasts’ molecular communication with hyperactivated myofibroblasts [2]. The cascade of these interactions is presumably initiated by a vascular injury caused by genetic or structural abnormalities, a hyperactive immune system or unknown external factors. The altered endothelial cells undergo pathologic interactions with fibroblasts and immune cells, which leads to a constant state of inflammation and fibrosis progression [2]. The autoantibodies are one of the factors promoting pathological mechanisms and are already present before the occurrence of the first symptoms.

Due to extensive fibrosis and vascular dysfunction, SSc causes various morphological and functional abnormalities in both the skin and internal organs. The most common symptoms of systemic sclerosis include thickening and hardening of the skin, particularly in the fingers, hands, arms and face. An early sign of SSc is the Raynaud’s phenomenon, which entails episodic color changes in the fingers and toes, triggered by cold exposure or stress stimuli. Gastrointestinal involvement can cause symptoms like heartburn, difficulty swallowing and reflux, while lung issues result in shortness of breath, coughs and reduced lung function. Joint pain, muscle weakness and digital ulcers are also frequently observed. Overall, systemic sclerosis may affect various organs, leading to complications such as kidney, pulmonary and cardiac involvement. The disease can also manifest as an overlap syndrome with other rheumatological diseases, such as rheumatoid arthritis or myositis [3].

Various immune cells are involved in SSc’s pathogenesis. Antinuclear autoantibodies are detected in up to 95% of patients suffering from the disease and can influence its clinical course [4]. Currently, the list of SSc-specific antibodies includes anti-centromere (ACA), anti-topoisomerase I (anti-topo 1) and anti-RNA polymerase III (anti-RNA pol III) antibodies [5]. There are also other types of rarer autoantibodies, which have a possible clinical association with the disease, such as antibodies against ribonuclear proteins (anti-U11/U12 ribonucleoprotein (RNP), anti-U1 RNP and anti-U3RNP) and antibodies against nucleolar antigens (anti-Th/To, anti-NOR 90, anti-Ku, antiRuvBL1/2 and anti-PM/Scl) [6]. Apart from their clinical significance, antibodies against nucleolar antigens cannot always be linked with ongoing pathology. Anti-Th/To or PM-Scl75 antibodies were reported to be found in 11.9% of healthy blood donors [7]. Different antibodies are linked to certain clinical characteristics, therefore identifying a specific antibody type is crucial for predicting possible organ engagement, prognosis and treatment [6]. 

The anti-Th/To antibodies are rarely seen in diseases other than scleroderma [8]. Among patients with SSc, they occur with a frequency of 3–6% and are associated mainly with the limited type of disease [9,10,11]. In this review, we take a look at the anti-Th/To antibodies and their role in the diagnosis, possible phenotype, prognosis and treatment response in scleroderma patients.

## 2. Molecular Characteristics of Th/To Antigens

**Figure 1:**
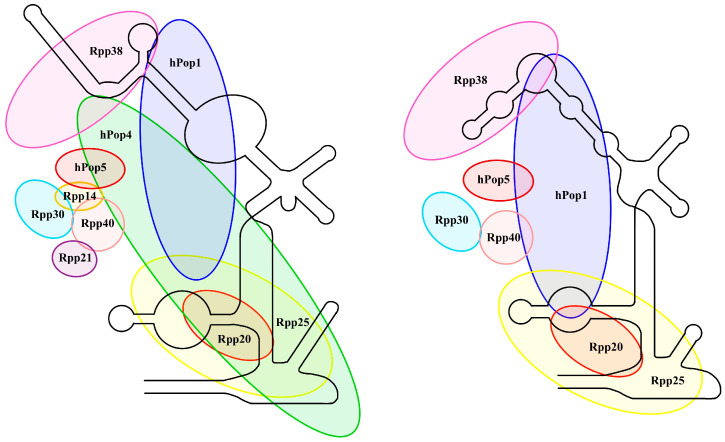
Structural features of RNase MRP and P complexes.

The Th/To nuclear antigens were first described in 1983 as 8-2 and 7-2 ribonucleoproteins, which are the components of RNase P and RNase MRP, respectively [12]. These protein complexes are mainly responsible for the maturation processes of different types of RNA [13]. They are composed of a non-coding RNA molecule and contain at least 10 protein subunits, see Figure 1 [14,15].

**Figure 2:**
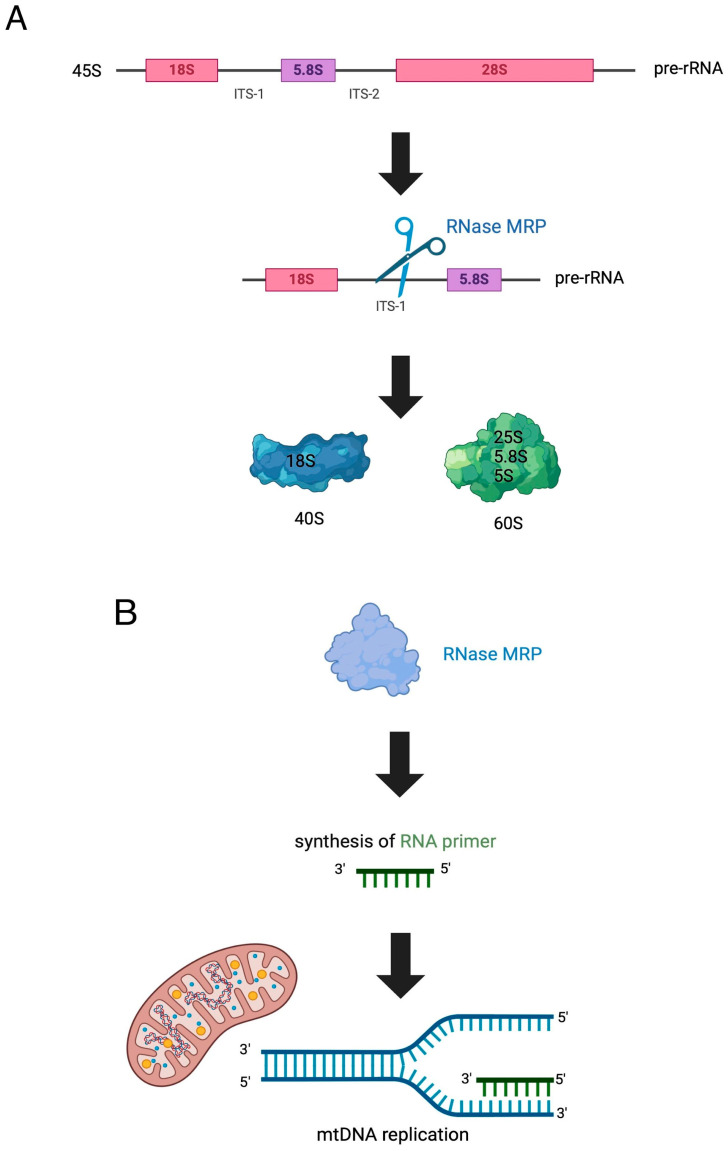

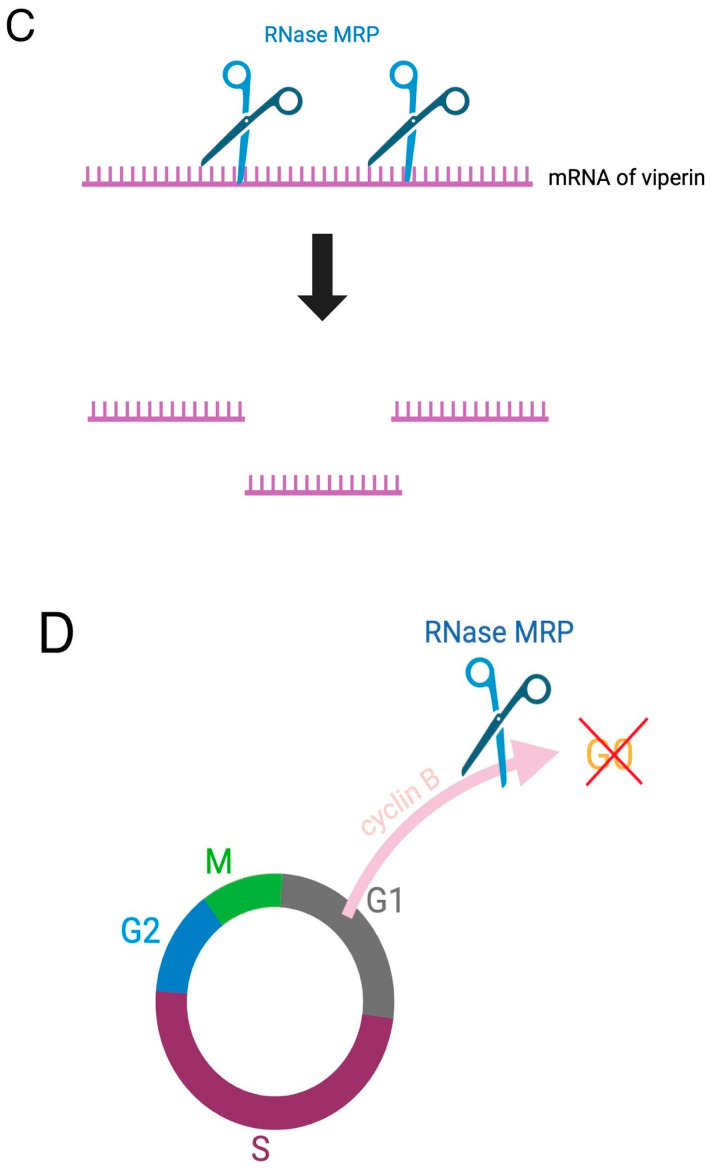
The function of RNase MRP in human cells [19]. (**A**) During ribosome biogenesis, RNase MRP participates in pre-rRNA processing. The cleavage takes place at the A3 site in ITS1 (internal transcribed spacer) and results in 25S, 18S and 5.8S rRNAs. Pre-rRNAs undergo essential folding, processing and modification to produce the pre-40S and pre-60S subunits. Once the last stages of maturation are completed, both subunits are exported to the cytoplasm, where they become ready for translation [20]. (**B**) RNase MRP generates RNA primers for mitochondrial DNA replication. (**C**) Viperin is an interferon-induced antiviral protein produced by human cells [21]. One of the functions of RNase MRP is to degrade viperin’s mRNA by cutting it at two cleavage sites [22]. (**D**) RNase MRP promotes the cell cycle via degradation of cyclin B mRNA, which, in its active form, stimulates the end of mitosis.

The essential function of RNase MRP in human cells is pre-rRNA processing [16]. It separates the 18S rRNA portion from the 5.8S–28S segment of the rRNA precursor, which constitutes a part of the internal transcribed spacer 1 [17]. This process contributes to the final product, which is the 60s ribosome. Another function of RNAse MRP is its impact on the cell cycle. The RNAse complex seems to degrade cyclin B2 mRNA, which is required to exit from mitosis [18]. All of the functions of the RNase MRP are shown in Figure 2.

Another ribonucleoprotein, RNase P, takes part in tRNA biogenesis. RNase P catalyzes the endonucleolytic cleavage of pre-tRNA and mediates the maturation of the 5′ end of tRNA [23]. Furthermore, it is involved in the transcription of RNA with the participation of polymerase I and III, which indicates RNAse P’s ability to coordinate gene transcription. The collaboration between RNase P and Polymerase III also seems to be involved in the innate immune system [24,25].

RNase MRP shares a degree of sequence homology with the RNA components of RNase P [26]. Moreover, most of the protein components of RNase MRP were also found to be identical to those in RNase P. At first, it was deduced that both complexes possess at least one common autoantigenic protein, Rpp38, which was named Th/40 as its molecular mass is 40 kDa [27]. Further investigations showed that most of the sera from anti-Th/To-positive patients contained autoantibodies that recognized mainly hPop1, Rpp30 and Rpp25 [13,28,29,30]. Knowledge of autoantigens can help to improve the diagnosis of scleroderma, as the sensitivity of commercial line immunoassay tests, also known as ENA tests, seems to be insufficient for the detection of anti-Th/To antibodies [31]. In a Mahler study from 873 ANA-positive patients suffering from SSc, extractable nuclear antigens remained undetected among 53 participants. Anti-Th/To, identified later by immunoprecipitation, were the most common and constituted 19/53 of the cases. The probable reason for the underdetection may have been the use of a different Th/To antigen in that assay. Therefore, it is essential to identify the protein subgroups of this antigen for accurate recognition.

Due to their intracellular localization, the antigens of RNase MRP and RNase P are inaccessible to the antibodies, making them unlikely to bind. However, in some circumstances, like massive cell death combined with impaired cell remnants clearance, it is supposed that antigens may be released, leading to immune complex formation and subsequent inflammation [32].

The role of anti-Th/To autoantibodies in the pathophysiology of these diseases remains unclear. Some SSc-specific antibodies, anti-Th/To, among others, stimulate endothelial cells and fibroblasts in vitro, suggesting a conceivable involvement in inducing a pro-inflammatory and profibrotic response [33,34].

## 3. Clinical Features

Between 92 and 99% of patients with anti-Th/To antibodies develop limited SSc [35,36]. In the study by Suresh, 97% of the patients, who were anti-Th/To antibodies-positive, had mild skin thickening, and in 23% of cases, systemic scleroderma sine sclerosis (ssSSc) was diagnosed [36]. ssSSc is a rare variant with absent or limited involvement of the skin with concomitant pathologic changes within the internal organs and serological findings [37]. The same variant was also found in patients from Fischer’s study, where ssSSc was the most common variant (69%) among a 13-person group with anti-Th/To antibodies, whereas the limited cutaneous subtype was detected in 31% of patients [38].

Comparing anti-Th/To patients with lcSSc to those that are ACA-positive, the first group has more subtle cutaneous and vascular involvement [39] and a mild clinical manifestation of the disease [40]. However, recent reports seem to deny the predisposition of anti-Th/To-positive patients to a mild course of the disease [36,39,41].

The first, and most common, co-occurring condition is interstitial lung disease (ILD), which develops in 18–54% of patients [35,36,39], and, alongside pulmonary arterial hypertension (PAH), is a recognized risk factor for increased mortality in SSc patients [42,43]. The profile of SSc-ILD varies widely, with less than a quarter of patients developing progressive fibrosing ILD, leading to respiratory insufficiency [44,45]. Due to their high specificity, anti-Th/To antibodies can serve as a reliable diagnostic marker for SSc-ILD [41]. While Mitri’s study showed that ILD is more common among Th/To patients compared to ACA-positive patients (48% vs. 13%), Moschetti et al. showed that anti-Th/To-positive patients developed ILD less frequently (40% vs. 85%) and required less immunosuppression (8% vs. 41%) than anti-topo I-positive patients [40].

The most common cause of death among anti-Th/To patients is pulmonary hypertension (PH), which develops independently of ILD [36,46]. In a study by Suresh et al., a 6.1-year clinical follow-up performed on 204 anti-Th/To antibody-positive SSc patients and 408 controls revealed that the presence of these antibodies leads to a significantly higher risk of developing PH, which stood at 38% among SSc-positive patients and 15% among negative ones [36]. The Mitri et al. study showed that PH was more common among anti-Th/To-positive patients compared to ACA-positive ones (28% vs. 18%, respectively) [39]. From the types of pulmonary hypertension that developed, pulmonary arterial hypertension was the most common and occurred in 23% of anti-Th/To antibody-positive and 9% of negative patients at the last follow-up visit. The other diagnoses were lung disease-related PH (13% of anti-Th/To antibody-positive and 5% of negatives) and cardiac disease-related PH (2% of anti-Th/To antibody-positive and <1% of negative patients). After adjustment for age and sex, the anti-Th/To-positive patients had a 3.3 HR (95% Cl 2.3–4.9; *p* < 0.0001) of developing PH within the next 10 years [36]. Other antibodies that have an important prevalence in SSc patients with PAH are antinuclear antibodies, anti-centromere and antiphospholipid antibodies and anti-U3 RNP antibodies, which appear in 80%, 50% and 25% of patients respectively [47]. In a 2018 review Nunes et al. noted that approximately 33% of anti-Th/To-positive patients suffered from PH and about 25% of patients diagnosed with PAH showed the presence of anti-Th/To antibodies [47].

The results of other studies also indicate a significant association between anti-Th/To antibodies and the presence of pulmonary hypertension in patients. PH occurred in following frequencies in anti-Th/To positive patients: 3/14 (23%) [48]; 4/4 (100%) [49]; 24/87 (28%) [39]; 32/72 (44%) [50]; 1/7 (14%) [51] and 3/8 (38%) [10]. In contrast, Kuwana et al. [52] reported no isolated pulmonary hypertension among five patients with anti-Th/To antibodies.

Scleroderma renal crisis (SCR) is a dangerous, life-threatening pathology that occurs in 2% to 15% of patients [53]. This complication predominates in diffuse scleroderma, while among patients with lSSc, it occurs with a frequency of 1–2% [54,55,56]. The development of SCR is primarily associated with anti-RNA pol III antibodies. [57]. As for anti-Th/To-positive patients, the risk is estimated at 3–5% [30,36,39]. In a study by Moschetti et al. [40], thirteen patients with anti-Th/To antibodies were evaluated. It was found that all of them presented limited cutaneous involvement and the Raynaud phenomenon. A noteworthy fact is that three-quarters of patients had esophageal symptoms, and half of them had digital ulcers and pitting scars. However, none of the patients with anti-Th/To antibodies developed PAH, synovitis or SCR, which argues in favor of a mild course of the disease. Also, none of the deaths among those anti-Th/To patients were caused by scleroderma-related conditions [40]. Moreover, the damage index (SCTC-DI) was low during follow-up. The survival rate in this cohort study at 5- and 10-years was 92.3% and 71.8%, respectively, and did not differ much from ACA-positive or anti-topo I-positive matched controls. Despite the favorable prognosis among patients who were anti-Th/To-positive, the SCTC-DI score showed a gradual increase over time. This was mainly due to the development of esophageal involvement (76.9%), digital ulcers (46.2%), sicca symptoms (38.5%), ILD (30.8%) and pericardial effusion (23.1%).

In the study by Mitri et al. [39], a comparison between anti-Th/To and ACA patients was also carried out. This study included a larger cohort of subjects, which consisted of 87 individuals with anti-Th/To antibodies. The anti-Th/To-positive patients presented with a shorter disease duration at initial prognosis compared to the ACA-positive patients and had more subtle cutaneous, vascular and gastrointestinal involvement [39]. SCR occurred exclusively among anti-Th/To patients. Also, an increased incidence of death from SSc-related diseases was found (72%) in those patients, predominantly associated with PAH. Similar findings were discovered in Ceribelli et al.’s study [9], where patients suffered from pericarditis (two out of eight patients with anti-Th/To antibodies) in addition to ILD and PAH.

Tendon friction rubs are a symptom described as a crepitus feel witnessed under palpation during active or passive movements. There is evidence which linked their presence to the diffuse disease type, shorter illness duration, reduced survival rate and involvement of the heart and kidneys [58]. In a study by Mecoli et al. [59] it was noted that, among 62 patients with Th/To complex, none of them exhibited tendon friction rubs, in contrast to the groups lacking these components, where 11% showed such a symptom (0% vs. 11%, respectively, *p* = 0.002). This suggests a lower likelihood of tendon friction rub development in individuals with these antibodies.

**Figure 3:**
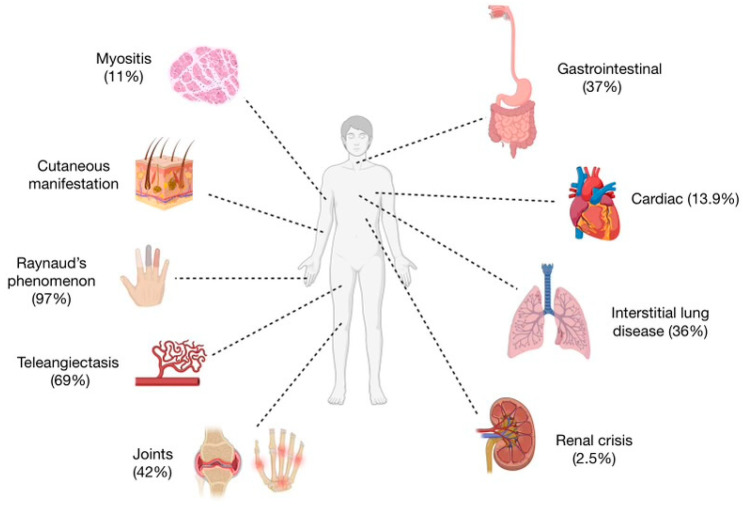
The clinical manifestation of anti-Th/To patients.

All connections between clinical profiles and the occurrence of anti-Th/To antibodies are shown in Table 1 and Figure 3.

The clinical profile seems to depend on different ethnicities. The Japanese patients tended to have less severely affected organs compared to the Caucasian ones [51]. Japanese patients with anti-Th/To antibodies showed rare internal organ involvement. The decrease in DLCO was also the lowest compared to other ANA antibodies and occurred among 29% of patients. Meanwhile, among 19 Canadian patients in Mahler et al.’s study, 70.7% showed a decrease in DLCO [31]. The findings suggest that these patients experience milder internal organ complications, leading to better survival rates compared to Caucasian patients with anti-Th/To antibodies.

There is a need for further research on systemic scleroderma, especially due to the small number of patients in the cohorts analyzed. Some studies conclude that anti-Th/To antibodies should be included in the routine clinical evaluation of individuals with scleroderma [31]. This is particularly crucial, considering they are the most frequently identified antibodies in SSc patients with false negative results for anti-nuclear antibodies, occurring in 19 out of 53 (36%) individuals who were ANA+/ENA− [31]. These antibodies have great potential as a valuable prognostic tool, aiding in a more precise diagnosis and treatment strategy. However, larger studies are needed to reliably assess their usefulness.

## 4. Raynaud’s Phenomenon

The incidence of the Raynaud’s phenomenon was relatively higher in SSc patients with anti-Th/To (80% vs. 56%) [36]. However, puffy fingers/hands and joint symptoms tended to be more frequent in SSc patients without those antibodies (respectively, 6% vs. 16%, and 2% vs. 11%).

In a prospective cohort study, 586 patients with Raynaud’s phenomenon were followed up for 20 years to specify whether autoantibodies and microvascular damage are predictive factors of progression to SSc [62].

At first evaluation, 80 (11.5%) of the 784 patients with Raynaud’s syndrome had SSc autoantibodies present, of which 12 (15%) patients had anti-Th/To autoantibodies. The following were recognized as independent predictors for microvascular damage: enlarged capillaries (HR 9.39, 95% Cl 5.43–16.25; *p* < 0.001), and later on in the disease progress, capillary loss (HR 2.4, 95% Cl 1.14–5.06; *p* = 0.022) [62].

Anti-Th/To antibodies were present in 13 of 74 patients (17.6%) who had abnormal findings during nailfold capillary microscopy at baseline and later experienced progression to definite SSc. After anti-CENP-B, which appeared in 33 (44.6%) patients, they were the second most commonly detected autoantibodies in this group of patients. Anti-Th/To antibodies had the hazard ratio of progressing to definite SSc at 5.9 in univariate analysis and 3.56 in multivariable analysis [62].

The temporal progression of capillary injury following the onset of Raynaud’s phenomenon in individuals with anti-Th/To antibodies exhibited a shorter duration compared to those with anti-CENP-B autoantibodies. However, it presented a longer duration compared to individuals with anti-RNA pol III autoantibodies [62].

## 5. Anti-Th/To Antibodies in Diagnosis of Scleroderma

In a 2023 study by Logito et al. [41], the anti-Th/To antibodies were reported to be 88.9% specific and 27.7% sensitive for SSc diagnosis. The positive predictive value (PPV) was 81.3%, and the negative predictive value (NPV) was 41.4%. The antibodies tended to be specific towards SSc-related ILD and were negative for non-ILD SSc patients, which makes it a valuable diagnostic clue for SSc-ILD [41]. The use of anti-Th/To and anti-fibrillarin together did not improve the specificity for SSc-ILD. However, the detection of anti-topo I still had the best sensitivity—85.1% (with a specificity of 19.2%, a PPV of 65.6% and a NPV of 41.7%)—compared to 27.7% for anti-Th/To and 12.8% for anti-fibrillarin autoantibodies. Together the three types of antibodies demonstrated a 95.7% sensitivity, 18.5% specificity, 67.1% PPV, and 71.4% NPV in the diagnostic procedure of SSc-ILD.

## 6. Inducing Profibrotic and Proinflammatory Response

In 2018, Raschi et al. [33] investigated whether immune complexes (ICs) containing scleroderma-specific autoantibodies can induce a profibrotic and proinflammatory response in skin fibroblasts, and the secretion of several substances is modulated by them. Following an incubation with ICs, fibroblasts were assessed for expression and secretion of different molecules that play an important role in three major processes in scleroderma etiopathology: inflammation involving IFNs, IL-6, ICAM-1 and MCP-1, vascular dysfunction characterized by IL-8 and ET-1 and fibrosis mediated by Pro-CollagenIα1 and TGF-β1. The results are shown in Table 2.

During the study, it was found that the fibroblasts’ response to anti-Th/To-ICs was mainly mediated by p38MAPK [33], which pathway stimulates fibrosis via TGF-β [63]. Supernatants obtained from the endothelial cells previously incubated with anti-Th/To-ICs caused a notable elevation in the α-SMA protein expression in skin fibroblasts [34], which is a structural protein involved in tissue remodeling [64].

**Table 2:** The effect on fibroblasts/endothelial cells obtained from healthy subjects after incubation with anti-Th/To-ICs based on studies of Raschi et al., 2018 [33], and Raschi et al., 2020 [34].

Mediators in Scleroderma	Fibroblasts after Incubation with anti-Th/To-ICs (Raschi et al., 2018) [33]	Endothelial Cells after Incubation with Anti-Th/To-ICs (Raschi et al., 2020) [34]	Role in SSc	Autor
IL-6	+	+	Induces expression of pro-collagen mRNA. Promotes fibroblast differentiation to myofibroblasts. In SSc it is associated with increased risk of PAH, ILD, cardiac and gastrointestinal involvement.	Kawaguchi et al., 2017 [65] Lin et al., 2022 [66] Zheng et al., 2023 [67]
IL-8	++	X	Chemoattractant for neutrophils, which are involved in the process of ILD. Elevated IL-8 levels are observed in patients with lcSSc, dSSc and with Raynaud’s syndrome. In SSc it is associated with Sjögren’s syndrome.	Crestani et al., 1994 [68] Reitamo, 1993 [69] Gourh et al., 2009 [70]
MMP-2	++	no data	Facilitates migration and invasion of endothelial cells into the surrounding tissue by degradation of basement membranes and extracellular matrix remodeling. Responsible for proteolytic processing of pro-inflammatory cytokines before their activation.	Waszczykowska et al., 2020 [71] Wen-jia Peng et al., 2012 [72]
MCP-1	++	no data	Role in infiltration of the skin by mononuclear cells and formation of inflammatory factors. Serum levels of MCP-1 were found to be increased in SSc patients with pulmonary fibrosis.	Distler et al., 2009 [73] Yamamoto, 2008 [74]
Pro-collagen type I alpha 1	++	no data	Activation of fibroblasts, resulting in excessive deposition of extracellular matrix, which mainly includes collagen I.	Manetti et al., 2017 [75]
TGF-β1	++	++	Responsible for vascular remodeling, indicating that TGF-β1 plays a role in the pathogenesis of PAH in SSc. Mediator of both fibrosis and vasculopathy.	Ayers et al., 2018 [76] Korman, 2019 [77]
α-SMA protein	no data	++	In SScα-SMA protein provides contractile force in stress fibers necessary for tissue remodeling, increasing connective tissue stiffness.	Manetti et al., 2017 [75]
ICAM-1	++	++	Proadhesive phenotype in SSc skin; induces myeloid cell adhesion to dermal fibroblasts. Leads to accumulation of leukocytes. Accumulation of lymphocytes T may contribute to fibrosis induction through the release of cytokines, which subsequently triggers excessive synthesis of the extracellular matrix. Increased serum levels of soluble ICAM-1 correlate with early stages of the disease and diffuse cutaneous SSc.	Rabquer BJ et al., 2009 [78] Abraham et al., 1991 [79] Sato et al., 1999 [80]
Et-1 mRNA	++	++	An endogenous vasoconstrictor, stimulates vascular wall cells’ proliferation, fibrosis and inflammation. A substantial correlation was observed between plasma levels of ET-1 and the quantity of digital ulcers and scars.	Cozzani E et al., 2013 [81] Aghaei et al., 2012 [82]
MMP-1 mRNA	X	X	Decreased level in SSc patients, in healthy individuals MMP-1 is responsible for the degradation of collagen.	Frost et al., 2012 [83]
TLRS				
TLR-2	++	X	Activates NFκB and stimulating the secretion of IL-6, which results in inflammation.	O‘Reilly et al., 2014 [84]
TLR-3	++	X	The function in the pathophysiology of SSc remains controversial: 1. This activation has been demonstrated to enhance the expression of TGF-β by fibroblasts, thereby playing a role in the overall fibrotic processes. 2. On the contrary, TLR-3 activation induces fibroblasts to produce IFN-I, which diminishes their capacity to produce extracellular matrix components.	Farina et al., 2010 [85] Fang et al., 2013 [86]
TLR-9	X	++	TLR-9 elicits fibrotic responses mediated by TGF-β1.	Fang et al., 2016 [87]

+—upregulation; ++—significant upregulation; X—no effect;PAH—pulmonary arterial hypertension; MMP-2—matrix metalloproteinase-2/collagenase type IV; MCP-1—monocyte chemoattractant protein-1; TGF-β1—transforming growth factor beta 1; α-SMA protein—alpha-smooth muscle actin; ICAM-1—intercellular adhesion molecule 1; Et-1—endothelin-1; MMP-1—matrix metalloproteinase 1; SSc—scleroderma; NFκB—anti-human nuclear factor kappa B.

Upon activation, endothelial cells play a vital role in the pathogenesis of the disease, facilitating the development of fibroproliferative vasculopathy [88]. This involvement is marked by an imbalanced production of vasoactive substances that induce vasoconstriction. The damaged endothelial surface exhibits an augmented expression of adhesion molecules, which enhances the migration, activation and accumulation of leukocytes within the vascular walls. Furthermore, endothelial cells undergo transdifferentiation into myofibroblasts, a process that contributes to vessel proliferation and occlusion. These events culminate in tissue hypoxia, further exacerbating cell injury and promoting the activation of fibroblasts [34].

The current study proposes that TLRs mediating the cellular response to SSc-ICs are located on the cell membrane rather than within intracellular compartments [34]. Additionally, endothelial cells reveal variations in TLR regulation when compared to previous findings in fibroblasts, indicating a cell-specific response to SSc-IC treatment, which is further supported by the different activation patterns of the intracellular mediators [34]. There is an increasing amount of evidence indicating that chronically activated TLRs play a harmful role in the development of SSc [84]. As for anti-Th/To antibodies, they induced a higher expression of TLR-9 on endothelial cells, which is a receptor known to be responsible for tissue fibrosis [89]. Stimulation with anti-Th/To antibodies led to the overexpression of TLR-2 and TLR-3 on fibroblasts. The involvement of both TLR-2 [84] and TLR-3 [90] in scleroderma is highly probable since SSc fibroblasts that overexpress these types of receptors produce an increased amount of IL-6, a crucial molecule in the fibrotic process.

## 7. Risk of Carcinogenesis

There are many studies investigating the correlation between cancer incidence and scleroderma. A comprehensive meta-analysis, based on data from six original studies, included 6641 patients diagnosed with scleroderma [91] and found that they are at elevated risk of developing malignancies, with an observed predilection for cancers of the lung, liver, bladder and hematopoietic system. The overall cancer risk defined by the pooled standardized incidence ratio was 1.41 (95% CI 1.18–1.68; *p* < 0.05) [91].

Therefore, many studies investigated the association between SSc and increased cancer incidence, suggesting the influence of chronic inflammation [92], fibrogenesis [93] and immunosuppressive therapies [94]. An association was also found between an increased incidence of cancer and the presence of antibodies, such as anti-RNA polymerase III autoantibodies [95].

While certain specificities of autoantibodies showed an increase in the risk of cancer, there is also evidence suggesting a potential protective role for other autoantibodies [96].

Both RNase MRP and RNase P (encoded by the RMPR gene) are essential for proper RNA processing. Defects in their activity are associated with SSc, malignant events, and connective tissue disorders [16,97]. Defects in the RMRP gene are the cause of diseases such as auxetic dysplasia, cartilage–hair hypoplasia and metaphyseal dysplasia without hypotrichosis [19,98,99].

In SSc, the most common autoantigens that anti-Th/To are directed towards are homologs of processing of precursor 1 (hPOP-1), ribonuclease P/MRP subunit p25 (RPP25), ribonuclease P/MRP subunit p40 (RPP40) and ribonuclease P/MRP subunit p30 (RPP30) [32]. Some of these seem to be linked to the increased occurrence of cancer.

It is worth noting that there are currently only a few studies, with small cohorts, that examined the relationship between tumor development and the presence of antibodies to Th/To antigen. Further studies should be conducted, including both gene expression and the simultaneous testing of antibody levels. This would enable a more comprehensive molecular assessment of the mechanisms involved in the development of cancer processes in patients with anti-Th/To antibodies.

Fan et al. found that hPOP-1 can be used as a prognostic marker of colorectal cancer [100]. Furthermore, it shows promise as a diagnostic and prognostic target for prostate cancer [101].

RPP25, a common SSc autoantigen, contributes to ribonuclease P activity via binding to H1 RNA [102]. According to Mahler et al., autoantibodies to the RPP25 are the most frequent ones in patients with negative tests for scleroderma [31]. RPP25 is a prognostic predictor of high-grade glioblastoma [103], the development of which is elevated among patients with scleroderma (HR 6.56 95% CI 1.64–26.21) [104]. It is supposed that this subunit of the Th/To antigen influences tumor progression by regulating cellular metabolism.

RPP30 is another common autoantigen in patients suffering from scleroderma. Individuals with positive anti-RPP30 antibodies are more likely to experience severe pulmonary manifestations and secondary pulmonary hypertension [47]. Under physiological conditions, RPP30 is responsible for the binding of H1 RNA to RNase MRP and the P complex [102]. The expression of Rpp30 is involved in the processes of breast and lung cancer development [105,106]. The upregulation of RPP40 is a poor prognosis biomarker of uterine corpus endometrial carcinoma [107]. Moreover, increased levels of RPP40 are implicated in chemoresistance in acute myeloid leukemia [108] and an upregulated expression of RPP40 mRNA predicts recurrence in early-stage triple-negative breast cancer [109].

Nevertheless, the positivity of anti-Th/To antibodies only represents the antigenicity of subunits of the Th/To autoantigen and does not indicate the abnormal expression or mutation of the RMRP gene. Further research into this is required. Various studies have proved that anti-Th/To autoantibodies are valuable tools in risk stratifying among patients with SSc for malignancy [59,110]. Currently, there is a lack of comprehensive clinical studies that would assess the risk of carcinogenesis based on particular SSc-autoantigen.

In a study by Mecoli et al., two cohorts of patients were compared. One group included patients with no cancer history 5 years after their initial symptoms of SSc and the other was composed of patients with a cancer history and coexisting SSc [59]. Anti-Th/To antibodies were detected equally often in both groups with the total frequency recorded as 8.3% of patients. However, the risk of developing cancer was reduced in the group of 67 patients with anti-Th/To antibodies (anti-hPOP1, -RPP40, -RPP30, and -RPP25 antigens) as compared to the other SSc patients without those antibodies (0% versus 11%; *p* = 0.009), which suggests their protective role in carcinogenesis.

Lung cancer and non-melanoma skin cancer were the most reported malignancies among anti-Th/To patients; but, in addition to cancer, these patients exhibited other risk factors such as smoking, ILD and immunosuppressive drugs [59].

Additionally, the authors indicated a potential protective effect of anti-Th/To antibodies, which appeared capable of decreasing the cancer risk associated with anti-RNA pol III positivity; it was also noticed that nine patients, who produced both anti-hPOP1 and anti-RNA pol III antibodies, did not have cancer-associated SSc within 3 years. In contrast, patients with anti-RNA pol III antibodies, but without anti-Th/To components, had a 2.18 times higher risk of developing cancer-associated SSc within 3 years compared to patients without anti-RNA pol III. However, further investigations on larger populations are necessary, as only nine patients in this study presented with both anti-Th/To and anti-RNA pol III antibodies [59].

Hoa et al. [60] also studied the prevalence and association between malignancy and the antibodies profile among SSc patients. A total of 29 out of 1682 patients had anti-Th/To antibodies and none of them developed cancer after 2, 3 and 5 years of SSc onset, indicating that individuals with anti-Th/To antibodies are not at risk of developing cancer due to SSc.

## 8. Presence in Other Diseases

Although anti-Th/To are primarily related to systemic sclerosis, their presence was noted among patients with other autoimmune diseases or a large autoinflammatory component. Their specificity to SSc was estimated to be 97% and their negative predictive value was 0.92 [7]. In research by Kuwana et al., they were found in patients suffering from rheumatoid arthritis, systemic lupus erythematosus, Sjögren’s syndrome, idiopathic thrombocytopenic purpura and polymyositis [8]. Koenig et al. also found them in autoimmune myositis [111]. They were reported to be associated with telangiectasis, sclerodactyly and scleroderma proximal to the metacarpophalangeal joints. However, the reactions were recorded to be “weak-positive” (11–25) in signal intensity units. Only a limited number of those reactions could be confirmed using immunodot assay (BlueDot) and fluorescence enzyme immunoassay (EliA). In the study, 285 patients with idiopathic pulmonary fibrosis were examined for the presence of ANA antibodies; 8.8% of them were found to be positive and 52% of this group consisted of anti-Th/To cases [38]. Four patients fulfilled three out of the five criteria for limited SSc, while nine patients met the criteria for SSc sine scleroderma. The prognosis of patients with ssSSc is estimated to be no different to that for those with idiopathic pulmonary fibrosis [38]. As there are no data about the effects of treatment regarding the specific sets of antibodies in SSc, this topic should be covered in future research.

## 9. Treatment

In the current recommendations for scleroderma management, the treatment targets are disease-modifying and organ-specific therapies [112]. Given the limited opportunities and unfavorable outcomes, there is a need for an enhanced understanding of SSc pathogenesis. The autoantibodies, including anti-Th/To antibodies, may act as the actual pathogenetic agents via releasing fibrosis and inflammatory cytokines [34]. Not only do they play a pivotal role in forecasting clinical phenotype and disease progression, but some studies also suggest their potential contribution to the treatment of scleroderma, involving targeting B cells or removing them from a plasma [113]. Other studies suggested TLRs as potential pharmacological targets, particularly during the initiation phase of the disease before the onset of overt fibrosis begins [114].

Currently, there is no specific recommended treatment for patients with anti-Th/To antibodies in SSc. However, autoantibodies may serve as guides in clinical treatment decision making. For example, the decision to initiate immunosuppression is likely to differ among patients with early ILD based on the presence of anti-Scl-70 antibodies, as these patients have a lower threshold for its introduction [115]. Caution is also crucial when introducing corticosteroids in early, diffuse SSc with anti-RNA pol III antibodies due to the high risk of SCR [116].

## 10. Conclusions

Although systemic sclerosis is a well-known medical condition that can be associated with a set of characteristic symptoms, it is still relatively poorly understood. One of the key components of its pathomechanism is a broad spectrum of autoantibodies such as anti-Th/To. Although highly specific to systemic sclerosis, they were also detected in patients with numerous rheumatoid diseases including rheumatoid arthritis, systemic lupus erythematosus and Sjögren’s syndrome. The antibodies target components of RNase P and RNase MRP which are involved in the process of RNA maturation. It was discovered that the presence of anti-Th/To antibodies can be associated with limited cutaneous involvement, pulmonary arterial hypertension, interstitial lung disease and an elevated incidence of Raynaud’s phenomenon. Nevertheless, plenty of fields still require profound research with much larger, diverse cohorts. To date, there are no prospective studies assessing response to treatment (especially targeted immunotherapy) based on specific types of antibodies, risk of carcinogenesis or correlation with other autoimmune diseases which can interfere with ongoing pathology. Moreover, it is necessary to increase the size of the cohorts in future studies in order to establish population-relevant patterns. Overall, pairing the antibodies with a specific clinical presentation, risk of complications and prognosis should be introduced as a clinical standard and another step in the advancement of the management of patients with systemic rheumatic diseases. Research in the field of antibodies and their relationship to specific treatment plans can eventually completely change our understanding of this and many other autoimmune diseases.

## Figures and Tables

**Table 1:** Characteristics of patients with anti-Th/To antibodies in overview studies.

Study	Moschetti et al., 2022 [40]	Suresh et al., 2023 [36]	Mitri et al., 2003 [39]	Charlton et al., 2017 [35]	Ceribelli et al., 2010 [9]	Mahler et al., 2014 [31]	Fischer et al., 2006 [38]	Hamaguchi et al., 2008 [51]	Hoa et al., 2021 [60]	Okano and Medsger, 1990 [48]	Graf et al., 2012 [10]	Höppner et al., 2023 [61]
number of all patients *	608	612	472	597	216	873	285	203	1698	371	129	372
th/to+ patients	13	204	87	199	8	19	13	7	29	14	8	14
mean age [years]		52.6	51.6		54.5	52.4			55			
women [%]	76.9	79	80	78	62.5	89.5	61.5	71	86		100	
mean age at ssc onset [years]	50		41.5	52.4	46	41		53	45.9		45.5	
smoking habit	7 [54%]	130 [64%]							17 [63%]			
Raynaud’s phenomenon	13 [100%]	202 [99%]	86 [99%]			19 [100%]	9 [69%]			12 [86%]		
duration of Raynaud’s phenomenon before SSc onset [years]	1		7.2		6							
limited cutaneous subtype	13 [100%]	198 [97%]	87 [100%]	197 [99%]	8 [100%]	17 [89%]		6 [86%]	22 [76%]	14 [100%]	6 [75%]	7 [50%]
mean mrss	3		4.1	2		5.7		7	8.3	6.4		
digital ulcers	6 [46%]		21 [24%]		4 [50%]	8 [42%]			17 [59%]			4 [29%]
pitting scars	6 [46%]		28 [32%]			8 [42%]		2 [29%]				
teleangiectasis	5 [38%]		70 [80%]		1 [12.5%]	14 [74%]	5 [38%]		20 [69%]	12 [86%]		
calcinosis	[8%]		13 [15%]		1 [12.5%]	6 [32%]	2 [15%]		7 [24%]	8 [57%]		
myositis	2 [15%]		5 [6%]			0		1 [14%]	4 [14%]	7 [50%]	1 [13%]	
articular involvement	0	109 [53%]	50 [57%]	62 [31%]		2 [11%]	8 [62%]	1 [14%]	9 [31%]	7 [50%]		
esophageal symptoms	10 [77%]		23/45 [51%]			11 [58%]	7 [54%]	2 [29%]		6 [43%]		
gastro-intestinalinvolvement	2 [15%]	109 [53%]	35/56 [62%]	26 [13%]						8 [57%]		
interstitial lung disease (HRCT)	4 [31%]	103 [50%]	33/68 [48%]	36 [18%]	3 [38%]	6 [32%]		2 [29%]	11 [38%]	6 [43%]	2 [25%]	6 [43%]
cardiac involvement	5 [38%]		12/57 [21%]	24 [12%]	2 [25%]	0		1 [14%]		0		
pulmonary arterial hypertension (group 1)	0	47 [23%]	24 [28%]	35 [18%]	0	0	5 [38%]	1 [14%]	3 [10%]	3 [21%]	3 [38%]	3 [21%]
renal crisis	0	6 [3%]	4 [5%]	3 [2%]		1 [5%]		0	1 [3%]	0	0	0
tendon friction rubs		6 [3%]	1 [1%]							0		
sclerodactyly						15/19 [79%]	0	3 [43%] **		3 [21%] **		

Values and percentages refer to the total number of patients with Th/To antibodies unless the number of patients is stated as x/y. In that case, the percentage is calculated from y. mRSS—modified Rodnan skin score; * also contains excluded patients; ** patient with joint contracture.

## Data Availability

No new data were created or analyzed in this study. Data sharing is not applicable to this article.

## Abbreviations

ACA	anti-centromere
anti-RNA pol III	anti-RNA polymerase III antibody
Et-1	endothelin-1
hPOP-1	homolog of processing of precursor 1
ICAM-1	intercellular adhesion molecule 1
ILD	interstitial lung disease
ITS1	internal transcribed spacer
lSSc	limited SSc
MCP-1	monocyte chemoattractant protein-1
MMP-1	matrix metalloproteinase 1
MMP-2	matrix metalloproteinase-2/collagenase type IV
NPV	negative predictive value
NFκB	anti-human nuclear factor kappa B
PAH	pulmonary arterial hypertension
PH	pulmonary hypertension
PPV	positive predictive value
RPP25	ribonuclease P/MRP subunit p25
RPP30	ribonuclease P/MRP subunit p30
RPP40	ribonuclease P/MRP subunit p40
RNP	ribonucleoprotein
SCR	scleroderma renal crisis
SSc	scleroderma
ssSSc	systemic scleroderma sine sclerosis
TGF-β1	transforming growth factor beta 1
anti-topo 1	the anti-topoisomerase I
SCTC-DI	the damage index
α-SMA protein	alpha-smooth muscle actin
